# Red blood cell phenotyping from 3D confocal images using artificial neural networks

**DOI:** 10.1371/journal.pcbi.1008934

**Published:** 2021-05-13

**Authors:** Greta Simionato, Konrad Hinkelmann, Revaz Chachanidze, Paola Bianchi, Elisa Fermo, Richard van Wijk, Marc Leonetti, Christian Wagner, Lars Kaestner, Stephan Quint

**Affiliations:** 1 Department of Experimental Physics, Saarland University, Campus E2.6, Saarbrücken, Germany; 2 Institute for Clinical and Experimental Surgery, Saarland University, Campus University Hospital, Homburg, Germany; 3 CNRS, University Grenoble Alpes, Grenoble INP, LRP, Grenoble, France; 4 Fondazione IRCCS Ca’ Granda Ospedale Maggiore Policlinico, Milano, Italy; 5 Department of Clinical Chemistry & Haematology, University Medical Center Utrecht, Utrecht, The Netherlands; 6 Physics and Materials Science Research Unit, University of Luxembourg, Luxembourg City, Luxembourg; 7 Theoretical Medicine and Biosciences, Saarland University, Campus University Hospital, Homburg, Germany; 8 Cysmic GmbH, Saarland University, Saarbrücken, Germany; Hebrew University of Jerusalem, ISRAEL

## Abstract

The investigation of cell shapes mostly relies on the manual classification of 2D images, causing a subjective and time consuming evaluation based on a portion of the cell surface. We present a dual-stage neural network architecture for analyzing fine shape details from confocal microscopy recordings in 3D. The system, tested on red blood cells, uses training data from both healthy donors and patients with a congenital blood disease, namely hereditary spherocytosis. Characteristic shape features are revealed from the spherical harmonics spectrum of each cell and are automatically processed to create a reproducible and unbiased shape recognition and classification. The results show the relation between the particular genetic mutation causing the disease and the shape profile. With the obtained 3D phenotypes, we suggest our method for diagnostics and theragnostics of blood diseases. Besides the application employed in this study, our algorithms can be easily adapted for the 3D shape phenotyping of other cell types and extend their use to other applications, such as industrial automated 3D quality control.

## Author summary

Microscopy offers the advantage of a direct visualization of the object under study. The observation of cell shapes can provide important information, such as the presence of a pathology. An application example relates to hematology, where the examination of blood smears gives first information on the diagnosis of a blood disease. At the same time, image analysis has been developing towards automation in order to provide objective, high-throughput and systematic results. Automated image recognition more and more tends towards sophisticated artificial-intelligence based methods. We here present a deep learning-based approach to classify the 3D shape of cells with accurate recognition of their fine surface details. Our system first performs a rough, discrete classification of cell shape, and second, a detailed morphological characterization by means of linear regression. Especially the latter task is impossible to be performed manually. We demonstrate the efficiency and the advantages of automated 3D shape evaluation over the traditional methods making use of 2D blood smear micrographs.

## Introduction

Cell morphology is a phenotypic characteristic reflecting the cell cycle, metabolic state or cellular activity [1–3]. While brightfield imaging is affected by the orientation of cells on the microscopy slide, which determines a certain projection, 3D confocal microscopy allows to investigate the whole cell surface without loss of information.

The analysis of shapes is related to feature detection in processed images. Machine learning-based approaches can potentially be employed for such tasks, avoiding manual procedures that are time consuming, subjective and prone to human error. Such type of approaches have been developed for 2D and 3D imaging for purposes ranging from pixel classification and object recognition [4, 5] to automated cell segmentation [6–9], reconstruction [10] and particle tracking [11]. Deep learning techniques have been recently explored based on multi-view projections [12, 13], but no methods have been employed for cell shape recognition of 3D confocal images. For this reason, we developed an artificial neural network-based method that provides a fine shape-detail recognition of 3D objects that are similar in shape and nature, thus having the potential to be universally used due to its precision. We demonstrate its efficiency for both classification and regression purposes. This method is implemented with a low computational cost based on 3D data reduction by extraction of the spherical harmonics spectrum. Recent studies corroborated the use of spherical harmonics descriptors to obtain an accurate cell shape reconstruction and classification [14–16].

To evaluate our system, we employed red blood cells (RBCs), representing one of the most deformable cell types. In healthy subjects, RBCs in stasis are typically biconcave disks, but external factors such as the pH or osmolarity of the suspension medium or interaction with surfaces can stimulate a shape transition. Such transformations appear in a distinct order and are described as the stomatocyte-discocyte-echinocyte (SDE) sequence [17]. In case of blood diseases, additional morphological abnormalities appear, defining certain blood disorders (e.g., hereditary spherocytosis, sickle cell disease, hereditary stomatocytosis or ellyptocytosis) [18]. The investigation of RBC morphology for the diagnosis of hematological diseases relies on the visual examination of blood smears. Advances in automation of the analysis have especially involved convolutional neural networks (CNNs) for white blood cell recognition [19] and, in some cases, for RBC detection and shape classification in 2D, both in stasis and flow [20–22]. However, in blood smear preparation the smearing and drying procedures affect cells, leading to unwanted morphological deformation [23] and loss of the 3D information of the original cell shape. Therefore, the substitution of the smear procedure with 3D imaging of suspended fixed RBCs combined with the automation of the analysis can lead to a significant improvement of the relevance of the results [24]. We here present a protocol to perform cell imaging, 3D reconstruction and related data transformation to feed the dual-stage artificial neural network (ANN). We provide results of the automated analysis of the 3D data on both healthy and diseased subjects affected by a genetic blood disorder, i.e. hereditary spherocytosis. This latter is caused by mutations in different genes coding for cytoskeletal proteins that can lead to severe anemia (for a detailed description, see S1 Text). The relation resulted between the mutation and the shape profiles suggests the use of 3D imaging for RBC phenotyping, with a potential application in diagnostics as well as in theragnostics to evaluate the success of a tested therapy.

## Materials and methods

### Ethics statement

Human blood withdrawal and handling from healthy donors and patients were performed with informed, written consent and in accordance with the Declaration of Helsinki and regulations and protocols approved by the ethic commission of the “Ärztekammer des Saarlandes” (reference No 51/18).

### Sample preparation for training data generation

Blood was drawn from healthy donors and patients via finger prick blood sampling in tubes containing 5 *μ*l of 1.6 mg/ml EDTA. For later imaging, 5 *μ*l of fresh blood from 10 volunteers and 10 patients with hereditary spherocytosis was fixed in 1 ml of either 1% or 0.1% glutaraldehyde in PBS (Gibco, USA) and stored at room temperature. Samples from patients were fixed and shipped from Fondazione IRCCS Ca’ Granda Ospedale Maggiore Policlinico of Milan (Italy) and University Medical Center Utrecht (The Netherlands).

Fresh blood from 5 healthy donors was employed to induce RBC shape transitions within the SDE scale by osmolarity variation of the suspension solution (S1 Fig). Ten microliters of blood was resuspended in 1 ml of NaCl solutions of different concentrations: 0.9% NaCl was used to preserve discocyte shapes and 0.4% NaCl was used to induce spherostomatocytes formation. Intermediate shapes were obtained by suspending cells in 0.5% NaCl (stomatocyte types I and II) and 2.5% NaCl (echinocyte types I and II) solutions (for echinocyte type III, see below).

A total of 400 *μ*l of each cell suspension was fixed in 1 ml of 1% glutaraldehyde (Sigma-Aldrich, USA) solution in NaCl. To fix cells with the desired shape, each glutaraldehyde solution was prepared to have a total osmolarity equal to the osmolarity of the NaCl solution used to induce each shape [25]. Fixed cells were placed in a rotator mixer (Grant-bio PTR-35, Grant Instruments, England) at room temperature overnight.

All fixed stored samples were prepared as follows. First, cells were centrifuged at 4000g for 5 minutes (Eppendorf Micro Centrifuge 5415 C, Brinkmann Instruments, USA), washed 3 times with 1 ml of each respective NaCl solution used to induce the different shapes and eventually resuspended in 1 ml of the same solution. Five microliters of CellMask Deep Red plasma membrane stain 0.5 mg/ml (Thermo Fisher Scientific, USA) was added to each sample for 1 hour at room temperature, followed by 3 washes. Echinocyte type III was formed with living cells by exploiting the glass effect. First, 10 *μ*l of blood was suspended in 1 ml of PBS and labeled with CellMask Deep Red, followed by 3 washes.

For each sample, cells were finally resuspended in PBS and immediately imaged.

### Imaging by confocal microscopy

Each labeled sample was placed between two glass slides for imaging (VWR rectangular coverglass, 24 × 60 mm) on top of a 60X objective (CFI Plan Apochromat Lambda 60X Oil, NA = 1.4, Nikon, Japan) of an inverted microscope (Nikon Eclipse Ti). A diode laser (λ = 647 nm, Nikon LU-NV Laser Unit) was used as a light source for imaging. Z-stack scanning was realized by employing a 300 nm piezo stepper for a 20 *μ*m z-range. Confocal image generation was performed with a spinning-disk based confocal head (CSU-W1, Yokogawa Electric Corporation, Japan). Image sequences were acquired with a digital camera (Orca-Flash 4.0, Hamamatsu Photonics, Japan).

### Image preprocessing

A custom written MATLAB (MathWorks, USA) routine was used to crop single cells from each image and perform their 3D reconstruction to enable visualization of the 3D shape. Each single-cell 3D image contained 68 individual planes with an extent of 100 px × 100 px and a lateral (x/y) detector resolution of 110 nm/px. The piezo stepper had a minimal step width of 300 nm, defining the z-resolution accordingly. Therefore, each obtained voxel had a box size of 110 nm × 110 nm × 300 nm. To compensate for the difference in resolution in the x/y and z directions, we adapted the scale in z by means of linear interpolation. Eventually, the obtained z-stack had dimensions of 100 px × 100 px × 185 px. The image stacks were then passed to a custom written ImageJ script. By applying a fixed threshold for every image, the script binarized the confocal z-stack to retrieve the cell membrane as an isosurface. After vectorization, the origin of the cell always corresponded to the center of its bounding box. Therefore, this step had the benefit of introducing an inherent translation invariance, i.e., for a given rotation, the form and size of the bounding box is invariant regarding translations in space. The obj-files (Wavefront Technologies, USA) generated in such a manner were then automatically transformed into the polygon file format (ply) and passed to the shape descriptor for the spherical harmonics analysis.

### Spherical harmonics analysis

The spherical harmonics analysis was performed by using the high-performance software implementation described by Kazhdan et al. [26]. The algorithm first maps a given 3D object (ply format) onto a 3D voxel grid of defined size. For our purpose, we kept the standard parameters of the algorithm, using a voxel grid of 64 px × 64 px × 64 px. Within this cube, at least 32 spherical functions of different radii can be arranged around the center point. Each spherical function can then be decomposed as the sum of its harmonics:

$$\begin{array}{c} f(\Theta ,\phi )=\sum_{l=0}^{\infty }\sum_{m=-l}^{m=l}a_{lm}Y_{l}^{m}(\Theta ,\phi ) \end{array}$$ (1)

Keeping the standard settings, for every radius, 16 frequencies (harmonics) were calculated. In addition, the first- and second-order components of each decomposition were expressed in a Euclidean manner using 3 scalars *a*_1_, *a*_2_, *a*_3_:

$$\begin{array}{c} f_{0}+f_{2}=a_{1}x^{2}+a_{2}y^{2}+a_{3}z^{2}. \end{array}$$ (2)

In the case of principal component analysis (PCA), these values could potentially be used for alignment purposes. However, in our case, these factors were not employed for further PCA investigations but were included in the training process. After transformation, each frequency component was accumulated by calculating the corresponding *L*_2_-norm. This resulted in a vector of size 32 × (3 + 14) = 544, where 32 corresponded to the number of radii, 3 to the scalars describing *f*_0_ and *f*_2_, and 14 to the number of remaining frequencies for each radius. Finally, each cell was expressed by means of a one-dimensional vector. For further signal processing, this vector was normalized and mapped onto a numeric range from 0 to 1. The intrinsic rotation invariance with respect to the original 3D data was the key factor in choosing this kind of transformation for shape description. This is because cells can have any orientation after sedimentation on the microscopy slide, and a general expression of their shape is required for further analysis.

### Manual classification

Each vectorized cell was rendered using Blender for cell shape visualization to perform a manual shape classification. The samples fixed in solutions at different osmolarities were used to classify the various shapes of the SDE scale based on Bessis’ criteria [27, 28]: (1) discocytes, meaning biconcave and symmetric disks; (2) stomatocytes type I and II, characterized by a lighter (I) and deeper (II) monoconcavity; (3) spherocytes, i.e., spherical cells; (4) echinocyte type I, crenated cells preserving biconcavity; (5) echinocyte type II, cells with forming spicules; and (6) echinocyte type III, cells with more than 25 spikes. Following the observation of other cell morphologies, the chosen shapes for additional categories were knizocytes, i.e. trilobal cells resulting from high shear stress or observed in some blood diseases; keratocytes, a category including damaged RBCs with variable shapes; acanthocytes, including echinocytes with irregular spicules and/or a spherical body; and multilobate cells, i.e., young reticulocytes. A class for the exclusion of an artifact occurring upon cell fixation, named cell clusters, was added. Each class contained a minimum of 10 cells to a maximum of 200 cells. Any other shape beyond the chosen classes was not considered and therefore not introduced in the training process.

### Training and validation of the dual-stage ANN

Keras with TensorFlow as the backend was used to build and train the dual-stage ANN. A set of representative RBC shapes was chosen for the supervised training of both ANN stages. Each of the selected cells was then transformed according to the previously discussed steps. In addition, data augmentation of the spectra was performed by creating 2000 linear interpolations between randomly picked spectra belonging to the same class, in the case of the first-stage ANN (classification) and between spectra of neighboring pseudodiscrete classes in the case of the second-stage ANN (regression) to cover the whole SDE shape spectrum. The augmentation also served to compensate for the different number of ideal cell shapes that were found for certain classes, ensuring a balanced training dataset (2000 total data per class). Similar to the k-fold cross validation approach, we trained both ANN stages with 100 different random starting conditions, finally selecting the best-performing ANN for each stage. The training data constituted 80% of the whole set of data, while the remaining 20% was used for ANN validation. The training was performed in batches of 100 spectra for both ANNs, finalizing the process at 100 and 40 epochs for the first-stage and second-stage ANN, respectively. The related loss functions were the crossentropy for the first and the mean squared error (MSE) for the second ANN, while the chosen optimizer for both was Adam [29].

### Automatic classification of 3D shapes in healthy individuals and patients

Samples from 10 healthy subjects and 10 patients affected by hereditary spherocytosis were automatically classified by the dual-stage ANN. The number of tested cells per sample ranged from about 1000 to over 2000 cells. The results were manually verified in Blender. Due to the large size of the whole dataset, in order to verify the automatic shape recognition accuracy a subpopulation of 2000 cells from 5 healthy donors and 5 patients (200 cells per donor) was chosen. The agreement between the predictions and the corresponding manual evaluation for each shape class was expressed in a confusion matrix.

## Results

**Fig 1:**
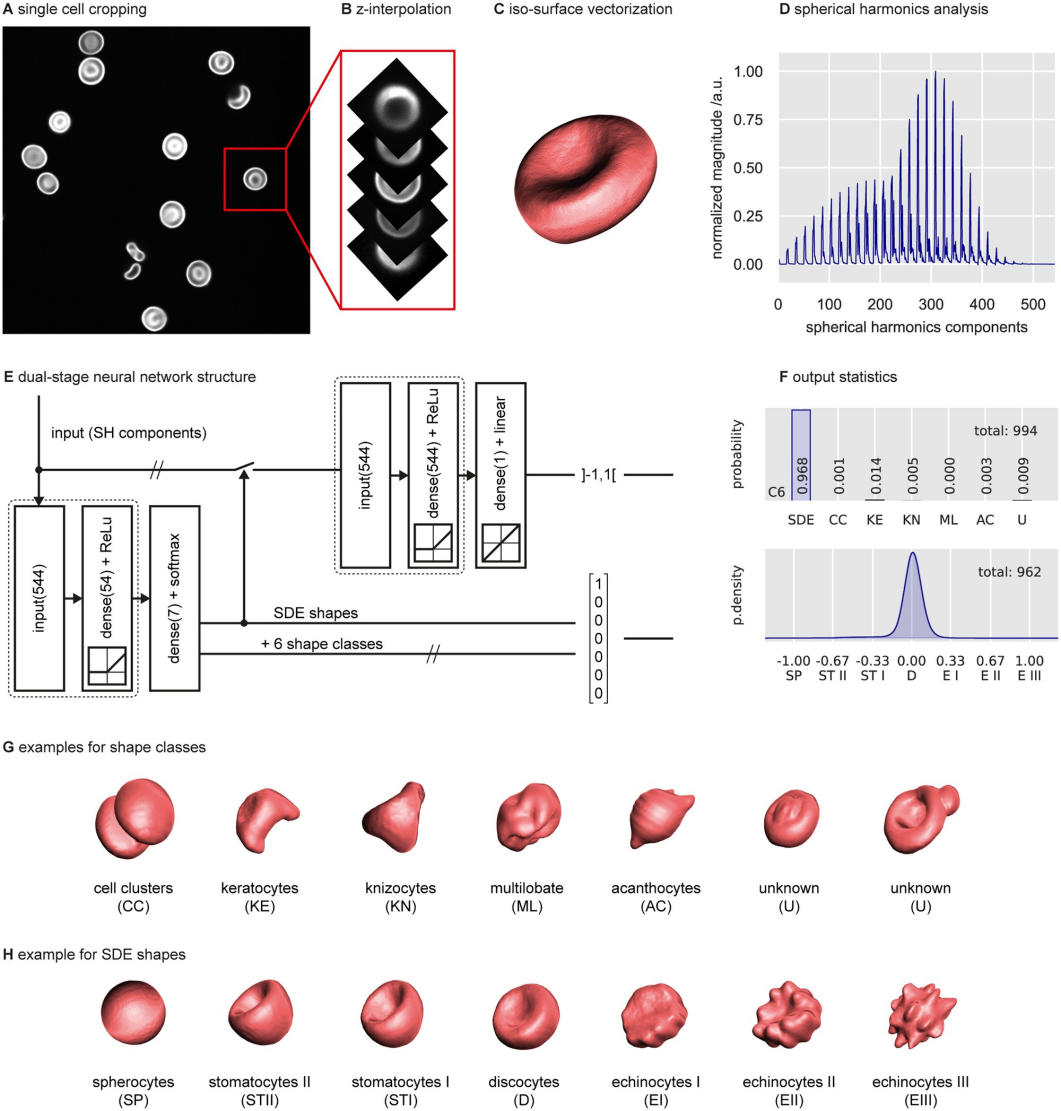
Workflow for automatic classification by the dual-stage ANN. (A) After sample staining and imaging by confocal microscopy, each cell is cropped individually and the full stack is interpolated in the z direction to achieve isotropic resolution (B). (C) The isosurface is retrieved by applying a constant threshold to each cell. (D) The vectorized data are transformed with respect to their spherical harmonics (*L*_2_-norm of 32 radii and corresponding 16 frequencies, see Materials and methods), representing a rotation invariant form of the cell shape. (E) Data are fed to the dual-stage ANN, with the first-stage resulting in a classification output (bottom). This stage consists of a three-layer architecture providing an input layer (544 neurons), a fully connected hidden layer with a ReLU activation function (54 neurons) and a fully connected output layer (softmax activation). The output is represented by a vector of size 7, which is subjected to discrimination by a given threshold. If the confidence is higher than 75% (threshold), the output is assigned to a certain existing class. Otherwise, the output is assigned to unknown (U) shapes. All detected SDE shapes are forwarded to the second-stage ANN, with an anatomy similar to that of the first ANN, except for the hidden layer that exhibits 544 neurons. The regression-type output layer assigns each cell a score between −1 and +1 and allocates them on the continuous SDE scale (top). (F) Example of a typical resulting shape distribution in a healthy subject. Almost all RBCs are discocytes. (G) Representative images for 6 mutually exclusive shape classes (see Materials and methods for description): two out of many different examples of “unknown” shapes are shown. (H) Representative cells of the SDE scale. The training data included SDE shapes artificially induced by changing the osmolality of the suspension medium.

After performing fixation of RBCs followed by fluorescent staining, confocal microscopy was used to capture the 3D representations of cells by means of z-stacks (Fig 1A). After offset elimination, intensity normalization and adaptation of the resolution in the x/y and z direction by interpolation (Fig 1B), we discriminated the cell membrane as an isosurface defined by a constant intensity threshold (Fig 1C). In contrast to preexisting classification approaches for such kinds of data, e.g., 3D-CNNs [9] or voxelwise processing techniques [13], we transformed and subsequently collapsed the volumetric data to exclusively access the features of interest. This data reduction was achieved by decomposing the spatial information of the cell surface into the respective spherical harmonics (SH) spectrum (Fig 1D and S1 Video) [26]. Thus, a one-dimensional data-vector was obtained, encoding the prevalent features of the 3D shape and characterized by rotation and translation invariance (see Materials and methods). A subsequent normalization mapped the SH spectrum into the range from 0 to 1, rendering the data suitable to train ANNs.

For cell shape recognition, we used a dual-stage ANN architecture (Fig 1E). The first stage was designed to sort out distinct RBC shapes that do not fit the SDE spectrum. Such shapes particularly occur in samples from patients with blood diseases or other pathologies [18, 30]. An additional class of “unknown” cells was added to reflect human uncertainty regarding unclear or rare shapes not yet defined in the literature. This class included all cells classified by the first-stage ANN with an identification accuracy below a threshold of 75%.

The second stage served to discriminate all SDE shapes. Previously, the SDE sequence was described by assigning different shapes to pseudodiscrete classes, such as spherocytes, stomatocytes type I/II, discocytes and echinocytes type I/II/III, to serve as reasonable support for manual classification [17]. By employing supervised training for both ANNs, we used the state-of-the art classification scheme to create training data of carefully selected sets of non-SDE (Fig 1G) and ideal SDE (Fig 1H) shapes.

In between the pseudodiscrete SDE classes, extra transition shapes were observed. For this reason, we assumed that the shape transformation occurs in a continuous manner and introduced a linear scale to automatically assign any identified SDE shape to an interval ranging from −1 (spherocytes) over 0 (discocytes) to +1 (echinocytes) (S1 Video). The regression-type ANN of the second stage allowed for fine distinction of morphological details within the whole spectrum at a precision that is manually unattainable.

The overall system consisted of (1) a classification-type ANN, which assigns each cell to one out of seven types (SDE shapes, knizocytes, keratocytes, acanthocytes, multilobate cells, cell clusters and unknown cells, Fig 1F top), followed by (2) a regression-type ANN, which characterizes all detected SDE shapes (spherocytes, stomatocytes type II/I, discocytes and echinocytes type I/II/III, Fig 1F bottom).

**Fig 2:**
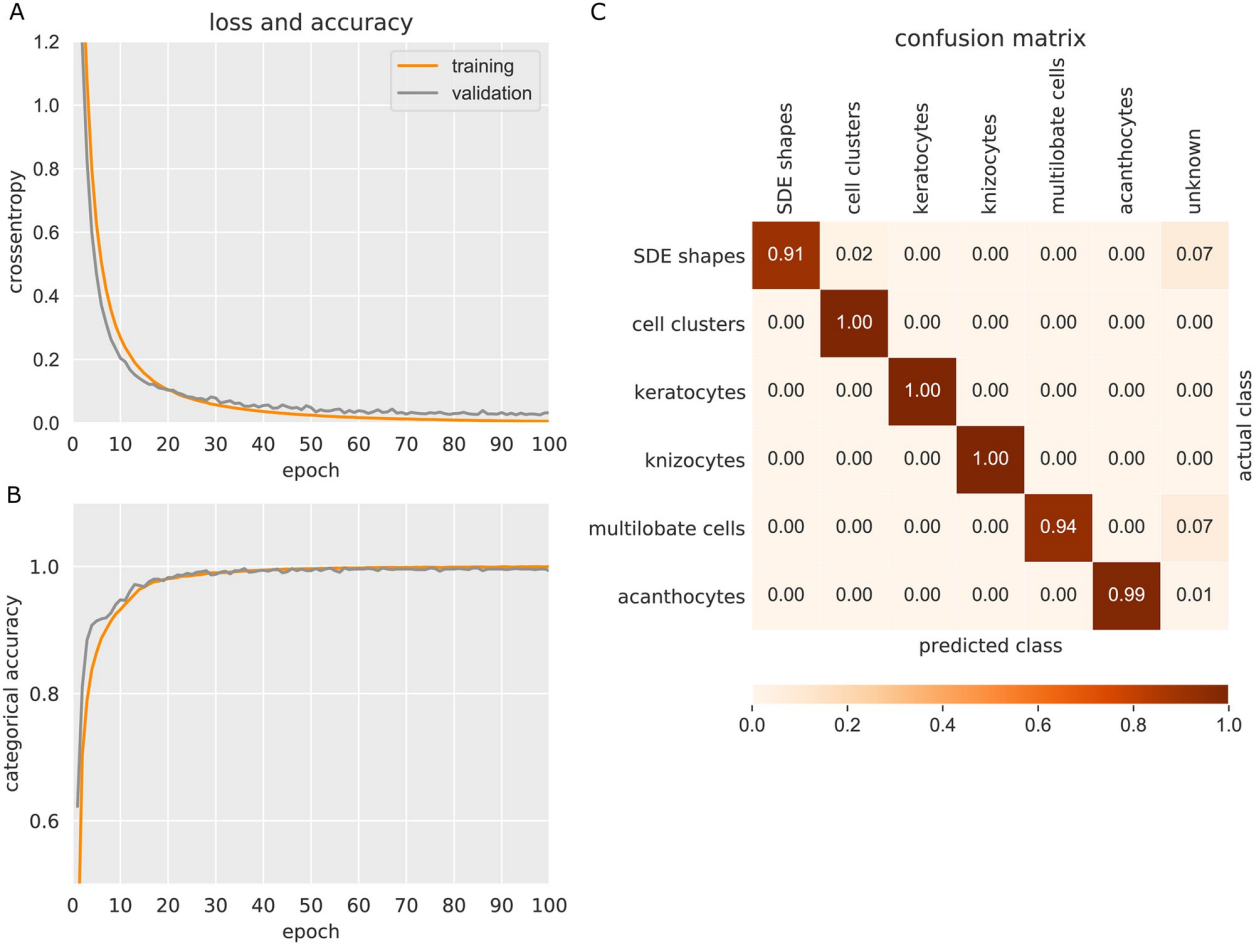
Training and validation loss and accuracy for the first-stage ANN. (A) During training, the employed loss function (crossentropy) was minimized throughout 100 epochs. (B) The categorical accuracy of cell classification for the training and validation sets converged close to 100%. The validation split was 20%. (C) The accuracy was further evaluated by means of a confusion matrix. “Predicted” versus “actual” (manually classified) cells demonstrate very good concordance, ranging from 91% to 100%.

**Fig 3:**
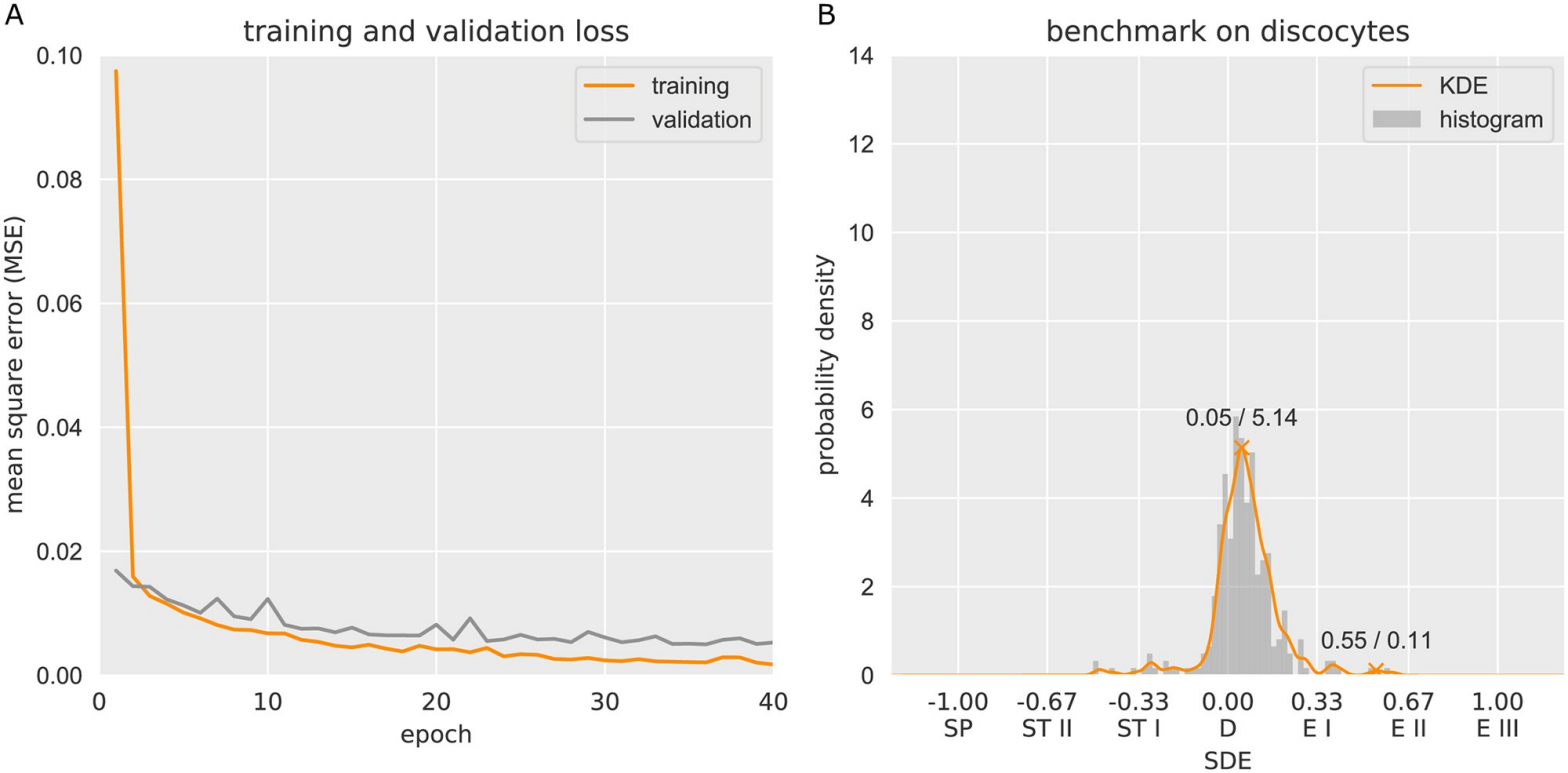
Training and validation loss for the second-stage ANN. (A) Within 40 epochs, the mean squared error (MSE) of the system was minimized. (B) Test on a representative set of 308 independent discocytes. The head of the distribution is allocated at 0.05, and the vast majority of cells is located within the range of −0.15 to +0.15.

The need for a vast amount of data for both training and validation was met by exhaustive augmentation. This was performed by selecting random pairs of cell shapes followed by random superposition and normalization of their spectra. In particular, we created artificial data within each of the mutually exclusive classes for the first-stage ANN as well as random interpolations between neighboring or the same SDE pseudodiscrete classes for the second-stage ANN. Avoiding a dependency between training and validation data, the split was performed before interpolation. The training and validation loss and accuracy for each ANN stage are shown in Figs 2 and 3.

**Fig 4:**
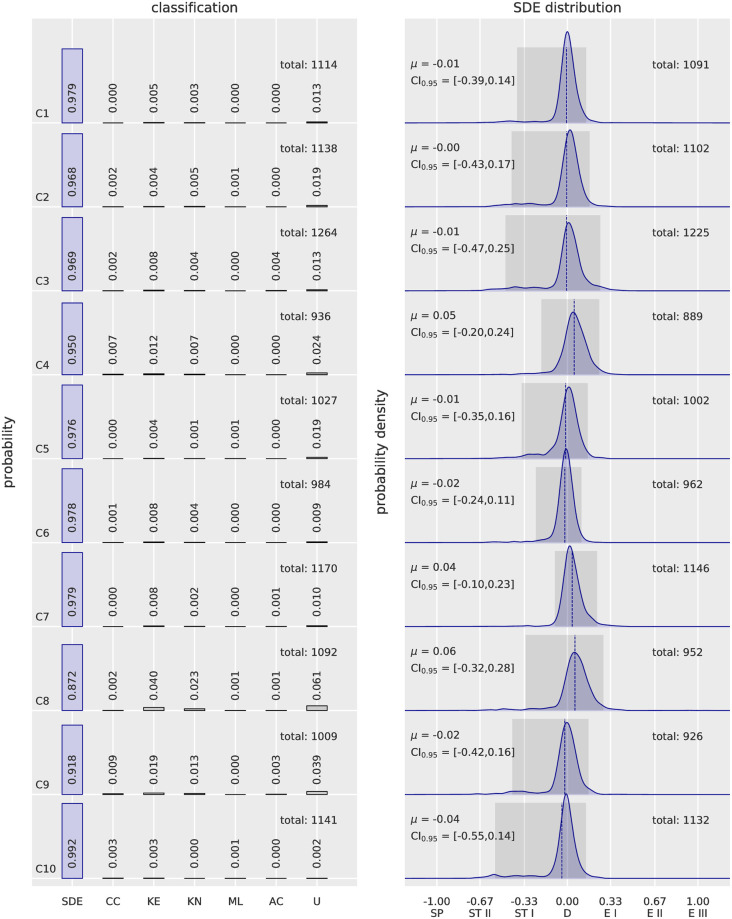
Automatic 3D shape recognition of RBCs from 10 healthy subjects. The total number of analyzed cells per patient is indicated on top right of the classification bar graph. As expected, in healthy individuals, shape distributions are centered around discocytes (0.00), with expected values *μ* (dashed line in diagrams) ranging from −0.04 to +0.06. The 95% confidence interval (CI_0.95_, dark grey area) demonstrates that shape distributions do not involve echinocytes, except for C8, which extends to the range of echinocytes I (+0.33). A very small amount of stomatocyte I exists in samples from most donors. The vast majority of cells are identified as “SDE shapes” (see total number in the classification panel versus total in the SDE distribution). The classification ANN contains a small percentage of cells classified as unknown. These results support the reproducible outcome of the dual-stage ANN analysis of RBCs from different donors and corroborate the differences seen between patients.

Our system was validated through the inspection of blood samples from 10 healthy donors (Fig 4), where a prevalence in discocytes is expected.

**Fig 5:**
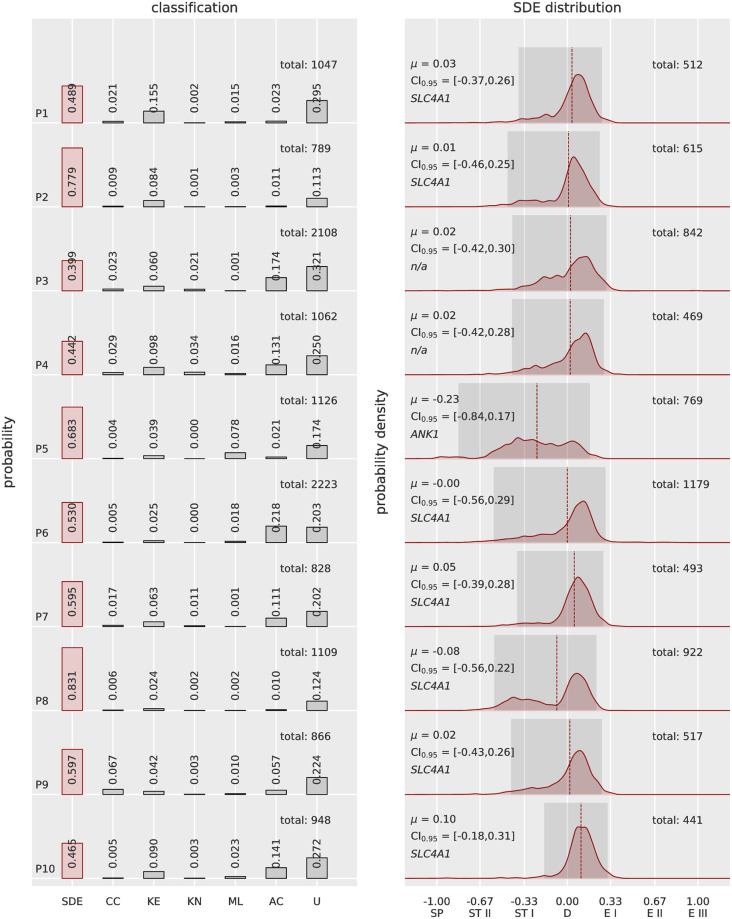
Automatic 3D RBC shape recognition for patients affected by hereditary spherocytosis. The total number of analyzed cells per patient is indicated on top right of the classification bar graph. The probability density distributions within the SDE range show the expected value *μ* (dashed red line) and highlight 95% confidence intervals (dark gray area). The results demonstrate that most of the patients have expected values related to discocytes, with a tendency toward stomatocytes. A population of spherocytes (score −1) is lacking in all 10 samples, proving that such a shape is not a hallmark of the disease. Additionally, shape profiles among patients are different, suggesting a relation to the various genetic mutations (*SLC4A1*, and in one case *ANK1*). In particular, P1 and P2 as well as P3 and P4 (nonidentified mutation) are relatives and show similar profiles, especially within the SDE range. P5 harbors a mutation that affects the cytoskeletal protein ankyrin, resulting in the highest number of stomatocytes, including some spherocytes. P6, P7 and P9 are affected by mutations in the band 3 protein, as is P8, who also has a double mutation in spectrin alpha (see Table A in S1 Text), although this latter mutation is not known to be pathogenic. The differences among these patients may depend on the particular mutation altering the same gene: P6 showed 22% acanthocytes, which occur in variable numbers in P1 and P2, P7 and P9. The phenotypic associated defect causes in some cases band 3 deficiency, while in others, it leads to spectrin deficiency (see Table A in S1 Text). Other classes showed rare occurrences, and shape deviations were classified as unknown cells in all the tested samples, suggesting that a larger amount of shape deformations was detected by the ANNs.

Then, the method was tested on 10 patients with different genetic mutations causing hereditary spherocytosis (*SLC4A1*, *ANK1*, Fig 5 and S1 Text and Table A in S1 Text). Results showed clear different shape profiles compared to healthy subjects, with a wider distribution in the SDE scale and a higher percentage of cells in other classes, including the class “unknown”. Of particular note, no spherocytes were found to be the major occurring shape (see S2 Fig). It is rather a prevalence of stomatocytes compared to healthy subjects a common feature observed in the patients. Overall, shape distribution profiles resulted in different classes abundance and variable SDE distributions. Considering the larger shape spectrum of patients, SDE distribution and the classification outputs resulted to be comparable (manual inspection) in the same subject’s samples fixed either in 0.1% or 1% glutaraldehyde when analyzing a total of about 800 or more RBCs (Figs 4 and 5). Interestingly, the relatives P1 and P2 as well as P3 and P4 showed respective comparable shape profiles, suggesting a possible relation between RBC shape deviations and genetic mutation.

**Fig 6:**
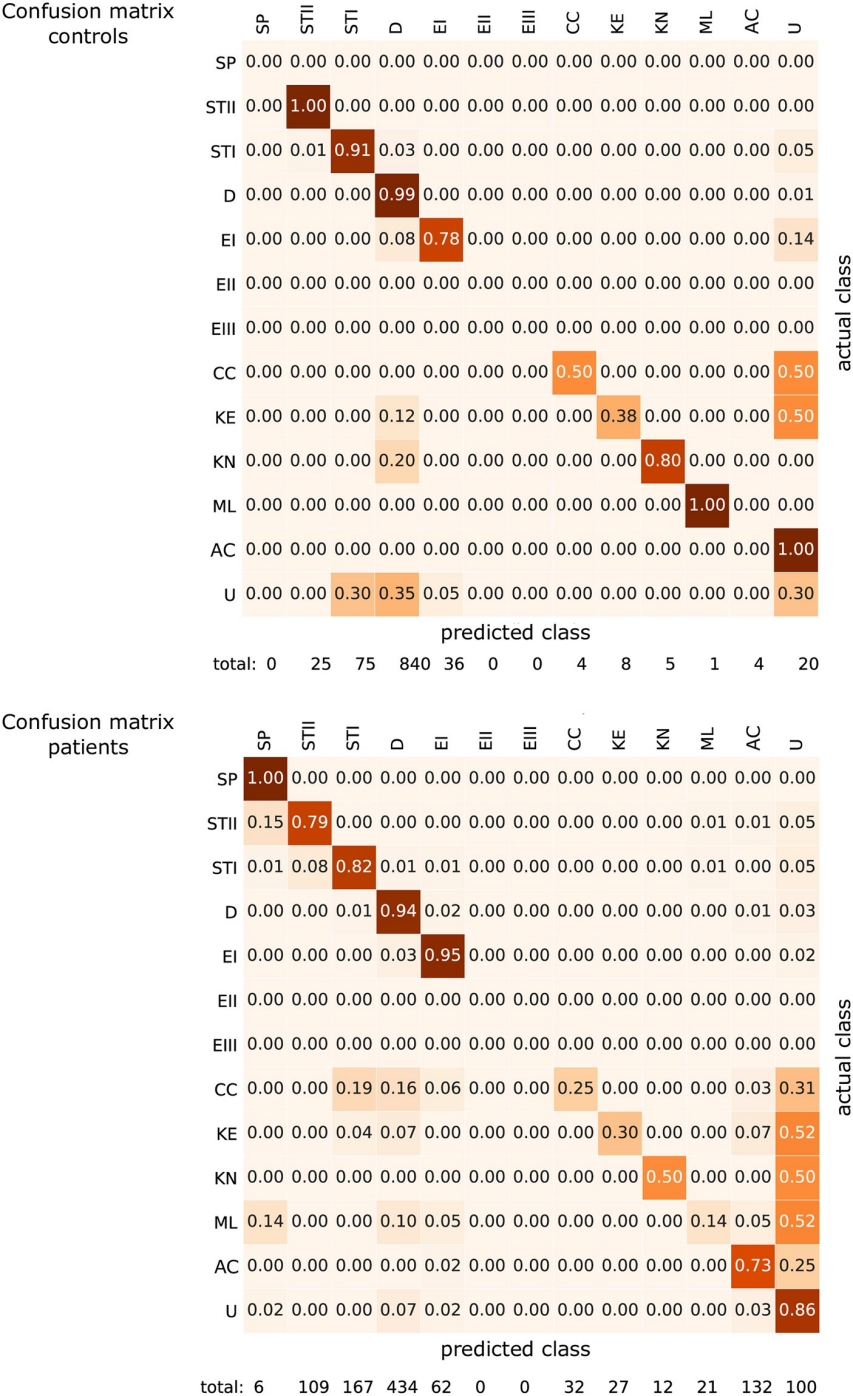
Confusion matrices for healthy controls and patients with hereditary spherocytosis comparing “predicted” (automated recognition) and “actual” (manually classified) shape classes. 2000 cells from 5 healthy subjects and 5 patients (10 donors × 200 cells) that were not included in the training process were randomly picked and the automated predictions of the dual-stage neural network was manually verified by observation of the corresponding 3D reconstruction. The confusion matrix shows the agreement percentage between the automated cell shape recognition (columns/predicted class) and manual inspection (rows/actual class). Since SDE shapes do not owe to a specific class, the accuracy of the predictions was judged by considering the interval between the score of two perfect shapes, which corresponds to ± 0.17. For instance, a discocyte (D) has score 0 and we considered correctly predicted any assigned value between 0 ± 0.17. The total number of cells encountered in each shape class during the manual verification is indicated. SDE shapes resulted in excellent recognition accuracy, ranging from 79% up to 100%. Cells belonging to specific classes showed a higher recognition error, partly due to the higher amount of unknown shapes, especially those occurring in patients, partly due to the less well-defined shape features that allow us to clearly distinguish each class. The observed error in such classes is related to the intrinsic inaccuracy of the training data. Echinocytes type II (EII) and III (EIII) correspond to 0% accuracy because they were not found in the dataset of randomly selected cells, thus resulting in no statistical relevance in all tested samples of healthy controls and patients.

As highlighted in a confusion matrix created for healthy controls and patients (Fig 6), the comparison of manual and automatic classification (first-stage ANN) resulted in a variable mismatch due to the rare occurrence of related shapes in blood samples and limited available training data. On the other hand, we observed a high agreement between the automatic allocation of cells on the SDE scale (second stage), ranging from 78% to 100%.

## Discussion

This study aimed at developing an automated tool for the analysis of cell shapes from 3D images. The preference of 3D is motivated by the impossibility of traditional 2D micrographs to identify the full surface details because of their intrinsic rotation dependence (see S2 Fig). In addition, the manual analysis causes high bias due to the lack of a quantitative measure to identify each cell shape in addition to the freedom of the observer to arbitrary exclude certain cells from the analysis. Conversely, an automated method indiscriminately takes into account the whole data, providing a truly reproducible and unbiased output.

Working with 3D images is challenging both for a manual and an automated analysis. Confocal imaging allowed us to obtain an optimal signal-to-noise ratio to define cell borders at fine detail, facilitating the manual classification, a necessary step to train the dual-stage neural network. However, the large amount of the 3D information and the variety of shape details of RBCs required a simplification of the 3D data to obtain an efficient training. The spectrum of shapes observed in 3D resulted wider compared to the classical definition of RBC shapes in literature, driving us to express SDE transitions onto a continuous scale. The transformation of the 3D data into a one-dimensional vector provided to be an excellent representation of cell shapes that could easily train the dual-stage ANN by accessing only the shape features of interest, instead of directly feeding it with the 3D images (e.g. 3D CNN), which include information on cell rotation and translation in the field of view.

From a clinical perspective, patients’ data resulted in a different statistical output compared to those of healthy donors. The tendency of RBCs to form a round shape is a hallmark of hereditary spherocytosis, and spherocytes are particularly expected in blood smears of affected subjects. A previous study reported that 2.6% of blood smears in a set of 300 patients did not exhibit detectable spherocytes, leading to a possible misdiagnosis [31]. However, the evaluation in 3D indicated that spherocytes in the tested set of patients were very rare or completely absent and comparable in number to those found in healthy subjects. These results confirmed the dependence of blood smear shape analysis on cell rotation, proving that spherocytes are not the main shape in hereditary spherocytosis.

The automated shape analysis was performed on a total cell number ranging from 800 to 2000 cells per sample. In the case of healthy subjects, the vast majority of cells is included in the SDE distribution and shows a peak in discocytes. The number of cells in the SDE distribution of patients is highly reduced because many are assigned to other classes. However, the larger confidence intervals in the SDE distributions of patients are not due to the lower number of cells included. The peak prominence between SDE distributions in patients with relatively low cell numbers (441, patient P10) is in fact comparable to others with a higher cell number (1179, patient P6), thus suggesting significant statistics in all the samples evaluated.

Some indications of the presence of other shape deformations in blood smears were reported [32, 33] and may have an association with the different genetic mutations causing the disease. The fine recognition of shape details by the automated dual-stage ANN resulted in a differential shape profile for various mutations. This represents additional information compared to that obtained from blood smears, where solely the type of blood disease can be discriminated. Finally, the prevalence of shapes occurring in non-SDE classes, especially in the unknown class, underlined the high morphological variability in patients, highlighting the demand for further RBC shape definitions.

## Conclusion

The application of our method in hematology revealed that conventional microscopy has limitations with regard to cell morphology that may lead to erroneous interpretations and shows the superiority of 3D visualization and characterization of cell shapes. While for other cell types, provided a sufficiently large training data, 2D imaging may be sufficient for a reliable shape recognition, the fine surface details determining RBCs shape variations require the 3D information. In addition to the unbiased outcome, automation by ANN allowed both the recognition of small shape details and the possibility of using a regression-based approach for cells undergoing continuous shape transitions. Owing to the details revealed using 3D imaging combined with ANNs as a universal tool for shape recognition, thorough tests on anemic subjects may render our method suitable for diagnostic purposes. In addition, from the results obtained with the tested pool of patients, we observed potential applicability with larger datasets to relate the ANN output to a particular mutation. Understanding the spectrum of RBC shape deviations in 3D in blood diseases will allow to test unsupervised clustering methods to distinguish different mutations. While genetic analysis is the gold standard for detection, cell imaging can be of additional interest in the investigation of the severity and state of a disease [34]. Moreover, it can be applied for personalized theragnostics when the effectiveness of a specific treatment is tracked [35]. Our method may be adapted for other cell biological applications or even industrial purposes.

## Supporting information

S1 Text Note on hereditary spherocytosis and tested patients. Table A. Information on the tested hereditary spherocytosis patients.
(PDF)

S1 Fig SDE training data were obtained by exposing RBCs from healthy donors to a different osmotic pressure. In isotonic solution, most of the RBCs are discocytes, (B). Upon decreasing the osmotic pressure (hypotonic solution), the RBCs exhibit swelling and transform into stomatocytes and further into spherocytes, (A). On the other hand, echinocytes develop in hypertonic solution, (C). Scale bar = 2 *μ*m.
(TIF)

S2 Fig Typical RBCs from 3 patients affected by hereditary spherocytosis. Each box shows three different rotations of the same cell; scale bar = 4 *μ*m. (A) RBCs from a patient with a mutation affecting ankyrin-1 and the respective blood smear in (B); scale bar = 10 *μ*m. While several spherocytes appear on the smear (arrows), 3D reconstructions show different kinds of shapes. Top boxes: mutated proteins are indicated. The top right box in each panel shows a “true” spherocyte from 3 different viewing angles. (C) and (D) are patients affected by other mutations. No “true” spherocytes were observed in 3D in (C). (D) Patient with a mutation in band 3, mostly showing stomatocytes rather than spherocytes.
(TIF)

S1 Video 3D reconstruction and corresponding spherical harmonics and shape allocation by the ANN in the SDE scale. The supplementary video shows a linear 3D morph between different RBCs considered to have perfect representative SDE shapes and belonging to specific positions within the SDE scale, (A). The morph was done to verify our system from a different perspective. While the ANNs were trained by superimposing the SH spectra of randomly picked pairs of cells from neighboring pseudodiscrete classes, the morph was carried out in the Euclidean space without loss of information. Every isosurface was first remeshed to obtain corresponding spatial coordinates. For each related grid-point, a stepwise linear interpolation was performed to pass from one shape to another. The whole set of obtained shapes was then transformed into the corresponding SH spectra shown in the video in (B), where characteristic details of each SDE shape can be observed. The related automatic allocation on the scale (yellow line) by the second-stage ANN was verified, (C). The gray lines highlight the previously recognized cells as histograms and demonstrate the continuous nature of the obtained shapes. The different cell counts at different positions are due to the stopovers at certain positions where the morph temporarily paused while image frames were consecutively created. The morph does not have the purpose of representing exact shape transitions as they occur in nature but exclusively aims to validate the linear interpolation approach of our system.
(MP4)
