# Supplementary material for: Red blood cell phenotyping from 3D confocal images using artificial neural networks
Source: PLoS Comput Biol. 2021 May 13;17(5):e1008934. doi: 10.1371/journal.pcbi.1008934 (PMC8118337; doi:10.1371/journal.pcbi.1008934)
Supplement: S1 Text — Table A. Information on the tested hereditary spherocytosis patients. (PDF) [file pcbi.1008934.s001.pdf]

Supporting Information

S1 Text. Note on hereditary spherocytosis and tested patients.

Hereditary spherocytosis is the most common hemolytic anemia in subjects of northern European ethnicity. This disease is mostly inherited in an autosomal dominant manner, although in approximately 20 % of cases, inheritance is autosomal recessive or due to *de novo* mutations [1]. The mutated genes code for RBC cytoskeletal proteins, most commonly the anion exchanger band 3 and the cytoskeletal protein ankyrin, as well as spectrin and proteins 4.1 and 4.2. Occasionally, patients are affected by a combination of multiple mutations in these genes. The defect mostly translates into a deficiency of the mutated protein. However, the genetic mutation may result in the deficiency of a different protein. An example is defects in band 3 and ankyrin occasionally causing the lack of spectrin integration in the membrane and degradation of free spectrin molecules, resulting in spectrin deficiency ([2, 3], **Table A in S1 Text**). Different mutations may occur in the same gene, leading to variations in disease severity [4].

Mutations in the abovementioned genes eventually lead to RBC membrane vesiculation that reduces the cell surface-to-volume ratio, transforming the RBC from a discocyte to a sphere-like shape [5]. These RBCs are named spherocytes and typically appear in blood smears as cells with a smaller and circular projected area, devoid of the characteristic central pallor observed in discocytes (S2 Fig). Such RBCs are less deformable, resulting in a reduced lifespan, which may eventually lead to anemia. This latter may be severe, moderate, mild or even absent when RBC loss is balanced by enhanced erythropoiesis.

Typical complications involve splenomegaly, reticulocytosis and hemolytic anemia, which can require exchange transfusions [6]. The only existing treatment is splenectomy, which improves cell survival and, reduces anemia, reticulocyte count and hyperbilirubinemia, but not the presence of spherocytes. Additional prophylaxis against infections is recommended [7].

The variable symptomatology and the numerous mutations make hereditary spherocytosis a highly heterogeneous disease. Moreover, spherocytes can be observed in other diseases [8] and can also be present as an artifact of the blood smear technique. Therefore, establishment of the correct diagnosis is dependent on several different tests.

For the 10 patients presented in this work, the diagnostic criteria were based on the following evaluations: presence of chronic hemolytic anemia, RBC morphology examination on blood smears, eosin-5'-maleimide (EMA) binding test, osmotic fragility test or altered osmotic gradient ektacytometry curve. Confirmation tests included sodium dodecyl sulfate-polyacrylamide gel electrophoresis (SDS-PAGE) analysis of RBC membrane proteins and next-generation sequencing (NGS) for mutation identification. The mutations and related detected protein defects are summarized in **Table A in S1 Text**. Patients P1 and P2 are relatives, as are P3 and P4. The latter were diagnosed with hereditary spherocytosis, but their mutation could not be detected. All patients were heterozygous for their main mutation. P8 was additionally found to be heterozygous for a point missense mutation **Table A in S1 Text** and homozygous for the α LELY low-expression alpha-spectrin variant. The missense mutation is considered a variant of uncertain significance (VUS), similar to homozygosity for LELY, but the latter may contribute to the eventual defective protein expression. The band 3 mutation is likely pathogenic and the main cause of the disease.

Table A in S1 Text. Information on the tested hereditary spherocytosis patients.

Patient	Mutated gene	Corresponding protein	Mutation	Phenotypical defect
P1	*SLC4A1*	band 3	c.2423G>A (p.R808H)	spectrin deficiency
P2	*SLC4A1*	band 3	c.2423G>A (p.R808H)	spectrin deficiency
P3	not identified	n.a.	n.a.	spectrin deficiency
P4	not identified	n.a.	n.a.	spectrin deficiency
P5	*ANK-1*	ankyrin-1	c.2559-2A>G (splicing)	predicted skipping of exon 26 and frameshift, loss or truncated ankyrin
P6	*SLC4A1*	band 3	c.620delG (p.G207fs)	loss or truncated band 3
P7	*SLC4A1*	band 3	c.2279G>A (p.R760Q)	band 3 deficiency
P8	*SLC4A1*, *SPTA1*	band 3, spectrin and α LELY	c.2279G>A (p.R760Q) c.3841C>T (p.R1281C)	band 3 deficiency
P9	*SLC4A1*	band 3	c.163delC (p.H55TfsX11)	band 3 deficiency
P10	*SLC4A1*	band 3	c.2510C>A (p.T837K)	band 3 deficiency

References

1. Bridges KR, Pearson HA, et al. Anemias and other red cell disorders/edited by Kenneth R. Bridges, Howard A. Pearson. New York: McGraw-Hill Medical; 2008.

2. Perrotta S, Gallagher PG, Mohandas N. Hereditary spherocytosis. The Lancet. 2008;372(9647):1411–1426. doi:10.1016/S0140-6736(08)61588-3.

3. Iolascon A, Avvisati RA. Genotype/phenotype correlation in hereditary spherocytosis. Haematologica. 2008;93(9):1283–1288. doi:10.3324/haematol.13344.

4. Dhermy D, Galand C, Bournier O, Boulanger L, Cynober T, Schismanoff PO, et al. Heterogenous band 3 deficiency in hereditary spherocytosis related to different band 3 gene defects. British journal of haematology. 1997;98(1):32–40. doi:10.1046/j.1365-2141.1997.1893005.x.

5. Bernhardt I, Ellory JC. Red cell membrane transport in health and disease. Springer Science & Business Media; 2013.

6. Hassoun H, Palek J. Hereditary spherocytosis: a review of the clinical and molecular aspects of the disease. Blood reviews. 1996;10(3):129–147. doi:10.1016/s0268-960x(96)90021-1.

7. Eber S, Lux SE. Hereditary spherocytosis-defects in proteins that connect the membrane skeleton to the lipid bilayer. In: Seminars in hematology. vol. 41. Elsevier; 2004. p. 118–141.

8. Deng Z, Liao L, Yang W, Lin F. Misdiagnosis of two cases of hereditary spherocytosis in a family and review of published reports. Clinica Chimica Acta. 2015;441:6–9. doi:10.1016/j.cca.2014.12.002.
