# Supplementary figures and images for: Red blood cell phenotyping from 3D confocal images using artificial neural networks
Source: PLoS Comput Biol. 2021 May 13;17(5):e1008934. doi: 10.1371/journal.pcbi.1008934 (PMC8118337; doi:10.1371/journal.pcbi.1008934)

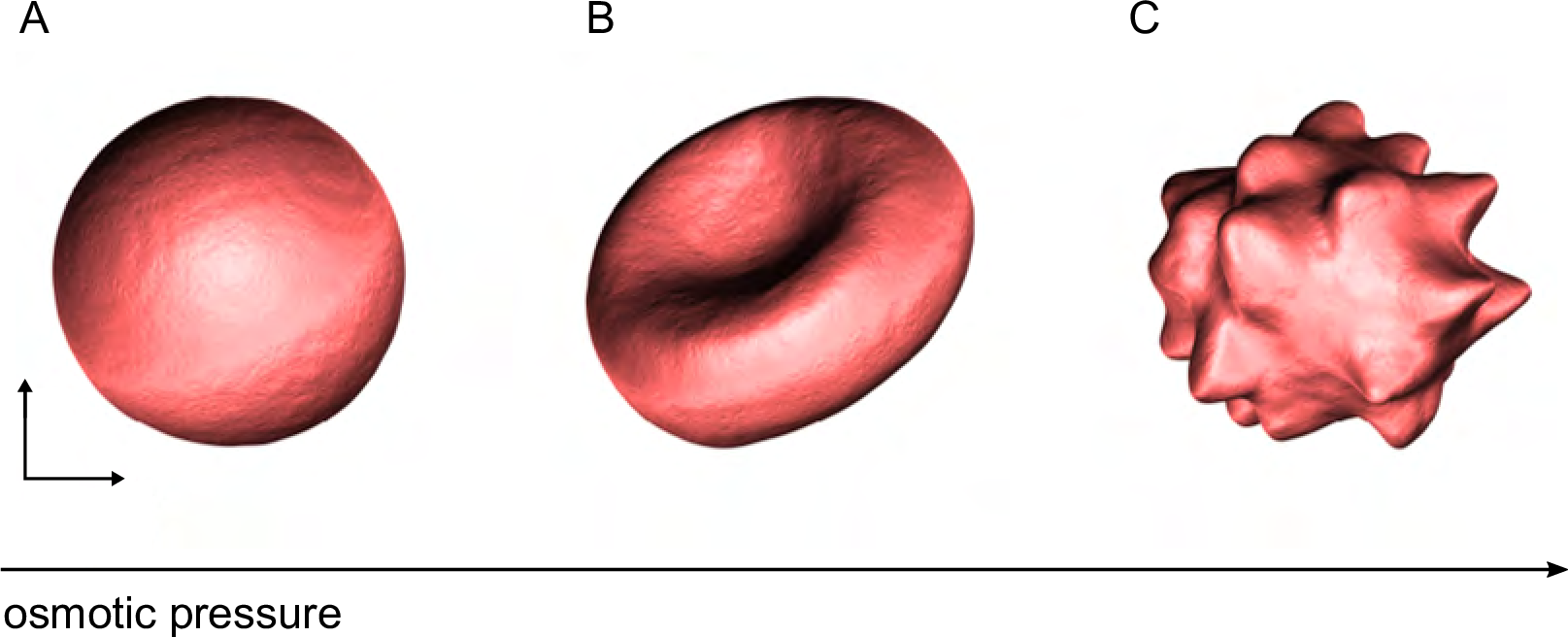

Supplement: S1 Fig — In isotonic solution, most of the RBCs are discocytes, (B). Upon decreasing the osmotic pressure (hypotonic solution), the RBCs exhibit swelling and transform into stomatocytes and further into spherocytes, (A). On the other hand, echinocytes develop in hypertonic solution, (C). Scale bar = 2 μm. (TIF) [file pcbi.1008934.s002.tif]

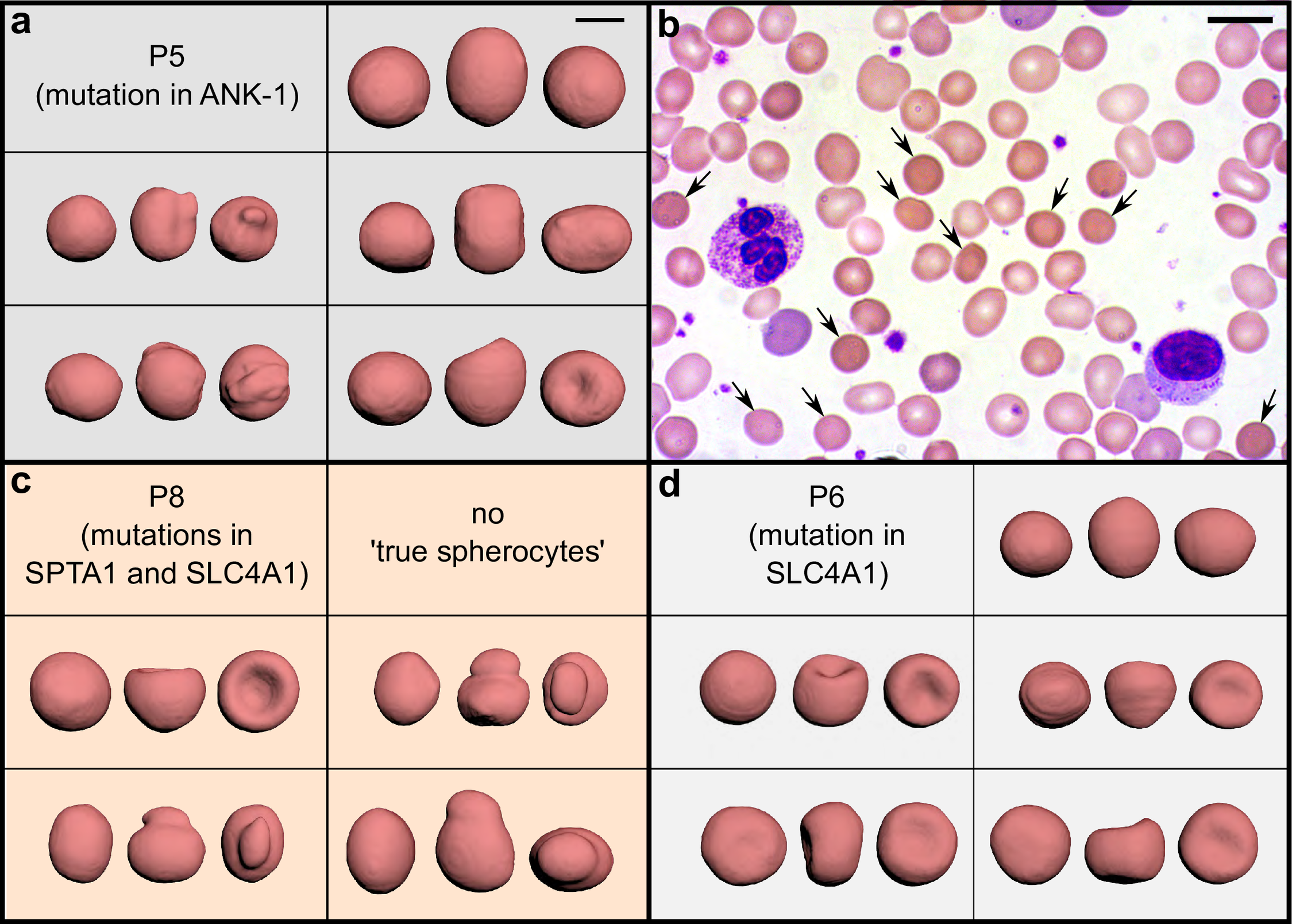

Supplement: S2 Fig — Each box shows three different rotations of the same cell; scale bar = 4 μm. (A) RBCs from a patient with a mutation affecting ankyrin-1 and the respective blood smear in (B); scale bar = 10 μm. While several spherocytes appear on the smear (arrows), 3D reconstructions show different kinds of shapes. Top boxes: mutated proteins are indicated. The top right box in each panel shows a “true” spherocyte from 3 different viewing angles. (C) and (D) are patients affected by other mutations. No “true” spherocytes were observed in 3D in (C). (D) Patient with a mutation in band 3, mostly showing stomatocytes rather than spherocytes. (TIF) [file pcbi.1008934.s003.tif]
